# In Vivo Regulation of Brain-Derived Neurotrophic Factor in Dorsal Root Ganglia Is Mediated by Nerve Growth Factor-Triggered Akt Activation during Cystitis

**DOI:** 10.1371/journal.pone.0081547

**Published:** 2013-11-26

**Authors:** Li-Ya Qiao, Sharon J. Yu, Jarren C. Kay, Chun-Mei Xia

**Affiliations:** Department of Physiology and Biophysics, Virginia Commonwealth University School of Medicine, Richmond, Virginia, United States of America; Institut National de la Santé et de la Recherche Médicale (INSERM U901), France

## Abstract

The role of brain-derived neurotrophic factor (BDNF) in sensory hypersensitivity has been suggested; however the molecular mechanisms and signal transduction that regulate BDNF expression in primary afferent neurons during visceral inflammation are not clear. Here we used a rat model of cystitis and found that the mRNA and protein levels of BDNF were increased in the L6 dorsal root ganglia (DRG) in response to bladder inflammation. BDNF up-regulation in the L6 DRG was triggered by endogenous nerve growth factor (NGF) because neutralization of NGF with a specific NGF antibody reduced BDNF levels during cystitis. The neutralizing NGF antibody also subsequently reduced cystitis-induced up-regulation of the serine/threonine kinase Akt activity in L6 DRG. To examine whether the NGF-induced Akt activation led to BDNF up-regulation in DRG in cystitis, we found that in cystitis the phospho-Akt immunoreactivity was co-localized with BDNF in L6 DRG, and prevention of the endogenous Akt activity in the L6 DRG by inhibition of phosphoinositide 3-kinase (PI3K) with a potent inhibitor LY294002 reversed cystitis-induced BDNF up-regulation. Further study showed that application of NGF to the nerve terminals of the ganglion-nerve two-compartmented preparation enhanced BDNF expression in the DRG neuronal soma; which was reduced by pre-treatment of the ganglia with the PI3K inhibitor LY294002 and wortmannin. These in vivo and in vitro experiments indicated that NGF played an important role in the activation of Akt and subsequent up-regulation of BDNF in the sensory neurons in visceral inflammation such as cystitis.

## Introduction

Irritation/inflammation of the visceral organs often alters the properties of primary afferent pathways, causing visceral hypersensitivity demonstrated as a reduction in pain threshold and/or an amplification of painful sensation. A number of mediators including cytokines, chemokines, and growth factors that are identified in visceral organs during disease states can act on the local sensory nerve terminals, leading to an increase in the excitability of the axonal terminal and sensory hypersensitivity [1,2,3]; the increase in the axonal terminal excitability, in turn, promotes neuropeptide expression in and release from primary afferent neurons at the peripheral terminals and increases local blood flow exacerbating the inflammatory process [4,5]. Specific to sensory neurons that innervate the urinary bladder, inflammation of the viscera in pathological states such as cystitis results in considerable plasticity of the neuronal cell bodies demonstrated as significant changes in the level of neuropeptides, ion channels, and receptors [6,7,8,9,10]. 

Among many neuropeptides expressed by sensory neurons, brain-derived neurotrophic factor (BDNF) generated by the neuronal somata influences synaptic efficacy in the spinal cord via anterograde transport and regulates spinal central sensitization [11,12,13,14]. Our recent study shows that blockade of BDNF action in the primary sensory pathway via intrathecal instillation of BDNF neutralizing antibody attenuates bladder hyperactivity in a model of colonic inflammation [15], suggesting a role of BDNF in the regulation of bladder sensory responses. The role of BDNF in mediating sensory sensitization is also observed in other systems including colitis-induced visceral hypersensitivity in response to colonic distention [16], peripheral inflammation-induced somatic pain [17,18], cancer-induced bone pain [19], and a variety of other systems [20,21,22,23]. 

Interstitial cystitis/bladder pain syndrome (IC/BPS) affects millions of people characterized by an abacterial infection of the urinary bladder. Biopsy analysis reveals that nerve growth factor (NGF) is elevated in the inflamed bladder and secreted into the urine [24,25,26,27], and is considered as a biomarker for IC [28,29]. BDNF is also found in the urine of patients with bladder disease [29,30]. In cyclophosphamide (CYP)-induced cystitis, intrathecal injection of either a general Trk receptor antagonist or a BDNF scavenger reduces bladder hyperactivity and also reduces spinal extracellular signal-regulated kinase (ERK) phosphorylation [31]. BDNF enriched in the sensory neuronal cell body in the DRG is able to undergo anterograde transport to the nerve terminals to either the peripheral organs or the spinal dorsal horn where its release can modulate the local physiology. 

The transcriptional and posttranslational regulation of BDNF is controlled by complex mechanisms. Several signaling pathways have been predicated to have a role in BDNF expression in culture. These pathways include Ca^2+^-dependent signaling [32,33,34] and mitogen-activated protein kinase pathway (MAPK) [35]. The PKA and CaMKIV pathways are also involved in the conditional regulation of BDNF expression examined in the amygdala [36]. In terms of BDNF expression in primary afferent neurons, we hypothesize that factors expressed in the peripheral organs may regulate BDNF expression through retrograde transport. NGF may play a role in regulating BDNF in sensory neurons in cystitis. This hypothesis is generated based on several measures: a) NGF is elevated in the urinary bladder in cystitis [24,37,38]; b) NGF possesses the property of retrograde transport by activating neuronal cell bodies through activation of MAPK and Akt pathways [39,40,41,42]; and c) in cultured DRG explants retrograde NGF is able to increase BDNF expression in the DRG neuronal soma [43]. Akt is traditionally considered as a survival factor targeting Bcl proteins, pro-caspase and Forkhead [44,45]. A recent new concept reveals that Akt also participates in the modulation of sensory activity by regulating TRPV1 activity [46]. In cystitis, inhibition of the Akt pathway reverses cystitis-induced bladder hyperactivity suggesting a prominent role of Akt in regulating bladder sensory activity [47]. 

Taken together, the present study is undertaken to investigate the endogenous pathways that mediate sensory activity involving NGF-triggered Akt activation in the DRG where it leads to BDNF up-regulation. The primary sensory projection of the urinary bladder in rat lies in the lower lumbar segment thus this study specifically addresses changes in the L6 DRG during cystitis and mainly focuses on mechanistic regulation of BDNF in L6 DRG in vivo. 

## Materials and Methods

### Experimental animals and ethics statement

Adult male rats (150-200 g) from Harlan Sprague Dawley, Inc. (Indianapolis, IN) were used. All experimental protocols involving animal use were approved by the Institutional Animal Care and Use Committee at the Virginia Commonwealth University (IACUC # AM10315). Animal care was in accordance with the Association for Assessment and Accreditation of Laboratory Animal Care (AAALAC) and National Institutes of Health guidelines. All efforts were made to minimize the potential for animal pain, stress or distress as well as to reduce the number of animals used. 

### Cyclophosphamide-induced cystitis

CYP (Sigma-Aldrich, St. Louis, MO) cystitis was induced in rats using the technique previously described [48]. Briefly, cystitis was induced in rats by injecting CYP intraperitoneally at a single dose of 150 mg/kg. Control rats received volume-matched injections of saline. All injections were performed under isoflurane (2 %) anesthesia. 

### Immunohistochemistry

Animals were deeply anesthetized with isoflurane (2–3%) and then underwent euthanasia via intracardiac perfusion with oxygenated Krebs buffer (pH 7.4; 95% O2, 5% CO2) followed by 4% paraformaldehyde. The L6 DRGs were identified and sectioned parasagitally at a thickness of 20 µm. DRG sections from control and experimental animals were processed with primary antibodies rabbit anti-BDNF (1:500, Santa Cruz Biotechnology, Inc., CA) or rabbit anti-phospho-Akt (1:500, Cell Signaling Technology Inc. Danvers, MA). In double immunostaining, we used mouse anti-phospho-Akt (1:400, Cell Signaling Technology Inc. Danvers, MA) co-staining with rabbit anti-BDNF, or rabbit anti-TrkA (1:750, Santa Cruz Biotechnology, Inc., CA) co-staining with sheep anti-BDNF (1:500, Millipore, Billerica, MA). These antibodies were used in our previous studies and their specificity had been evaluated [12,15,48,49]. Tissues from all groups of animals (control and experimental) treated at the same time block were processed simultaneously. 

DRG cells with visible nuclei were counted with a Zeiss fluorescent photomicroscope. BDNF and phospho-Akt cell profiles were counted in 6 to 10 sections randomly chosen from each L6 DRG. The area of section containing cells (excluding the area containing fibers) was selected using free-line tools integrated with the AxioVision measurement software (Carl Zeiss, Inc.) and was measured as mm^2^. The number of positively stained cells was normalized against the measured area and expressed as number of cells per mm^2^. To avoid double counting, we chose every third section for one specific antibody stained. 

### Western blot

Freshly dissected L6 DRGs were homogenized in solubilization buffer containing 50 mM Tris-HCl, 150 mM NaCl, 1 mM EDTA, 1 % Triton X-100, 100 mM NaF supplemented with protease inhibitor cocktail (P8340, 1:100, Sigma-Aldrich) and phosphatase inhibitor cocktail 1 (P2850, 1:100, Sigma-Aldrich). The homogenate was centrifuged at 20,200 g for 10 min at 4 °C, and the protein concentration in the supernatant was determined using Bio-Rad DC protein assay kit. Protein extracts were then separated on a 10 % SDS-PAGE gel and transferred to a nitrocellulose membrane. The membrane was blocked with 5 % milk in Tris-buffered saline for 1 hour and then incubated with phospho-Akt (1:1000, Cell Signaling Technology Inc. Danvers, MA) antibody followed by horseradish peroxidase-conjugated secondary antibody. The bands were identified by ECL. For internal loading control, the same membrane was stripped and re-probed with antibody against the non-phosphorylation form of Akt (1:1000, Cell Signaling Technology Inc. Danvers, MA). The ECL-exposed films were digitized, and densitometric quantification of immunoreactive bands was performed using the software FluorChem 8800 (Alpha Innotech, San Leabdro, CA). The level of the phospho-bands was normalized with the level of non-phospho-bands obtained using the same membrane. 

### Quantitative real-time PCR

Total RNA was extracted using a RNA extraction kit RNAqueous (Ambion, TX). RNA concentration was determined spectrophotometrically. cDNA was synthesized using Cloned AMV First-Strand Synthesis Kit (Invitrogen) with random hexamers. Following reverse transcription, quantitative real-time PCR was performed for type I collagen with a Taqman probe mixed with PCR Master-Mix for 40 cycles (95 °C for 15 sec, 60 °C for 1 min) on a 7300 real-time PCR system (Applied Biosystems). Quantitative real-time PCR of the same sample was performed for β-actin expression as internal control. The level of BDNF mRNA was normalized against β-actin expression in the same sample that was calculated with ΔCt method. The expression level of BDNF in control group from each independent experiment was considered as 1, and the relative expression level of BDNF mRNA in experimental animals was adjusted as a ratio to its control in each independent experiment and expressed as fold changes (2^-ΔΔCt^-fold).

### DRG-spinal nerve two-compartmented culture

We used a Campenot chamber and a modification of the method of Delcroix et al [42] to study the DRG-spinal nerve complex. The Campenot chamber has two large compartments on both sides and a narrow one in the middle. After the chamber was sealed onto the bottom of a cell culture plate, the middle compartment was filled with 1 % agarose serving as a divider and the two large compartments containing Dulbecco's Modified Eagle Medium (DMEM) were used for separation of the ganglion and nerve terminals of the isolated L6 DRG-spinal nerve complex. To test whether there was a leak between compartments, we initially filled one of the compartments with DMEM and watched for pass-through of the DMEM to the other compartments. When no sign of leakage was present, DMEM was added to the other compartment and tissue was then placed into the chamber. 2-3 layers of small filter paper soaked with DMEM were lightly placed on top of the nerve segment that crossed over the center compartment (“bridge”). The culture plate was then placed into cell culture incubator at 37°C. After 3 h of quiescent time, the nerve terminals were treated with NGF. 

### Drug treatment

To block the PI3K/Akt pathway in vivo, an intraperitoneal injection of a PI3K inhibitor LY294002 (Calbiochem, dissolved in DMSO as stock and diluted in saline for injection) at a single dose of 50 µg/kg body weight was made immediately after CYP injection. The same amount and concentration of DMSO was used as vehicle control. To block NGF action in vivo, a NGF antibody or control IgG (Santa Cruz Biotechnology, Inc. Santa Cruz, CA) was injected intraperitoneally at a dose of 30 µg/kg body weight according to previously published protocol [48]. A single dose of NGF antibody or control IgG was made immediately after the CYP injection. Both LY294002 (1 µM) and Wortmannin (0.5 µM, Calbiochem) were used in culture to block the PI3K/Akt pathway. 

### Statistical analysis

The results from each study were presented as mean ± SE. Comparison between control and multiple experimental groups was made by using a one-way ANOVA followed by Dunnett’s test. When two groups were compared, a *t*-test was used. GraphPad Prism 5 was used for analysis. Differences between means at a level of p≤0.05 were considered to be significant. 

## Results

### Cystitis-increased BDNF mRNA and protein levels in L6 DRG is regulated by endogenous NGF

**Figure 1:**
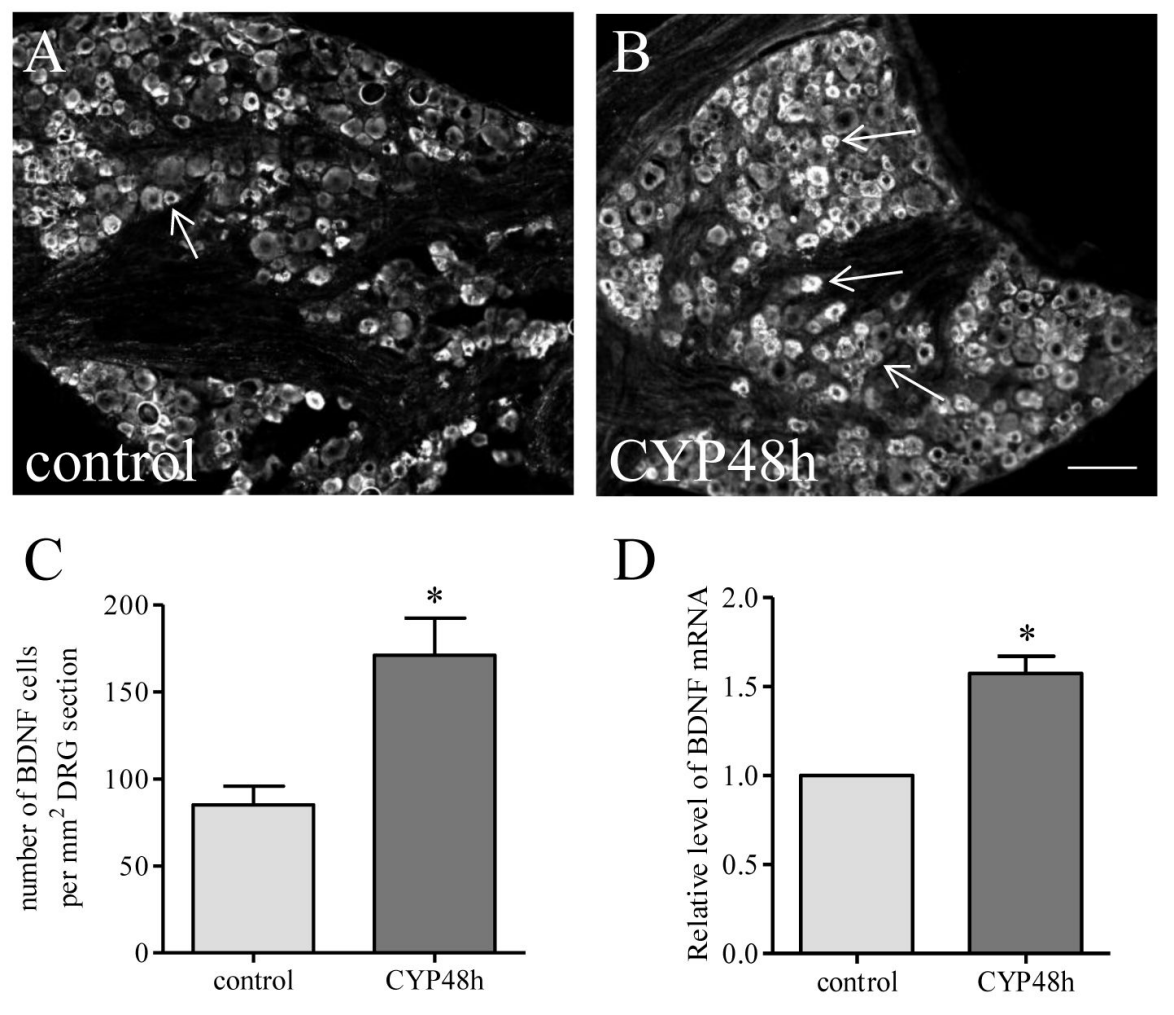
Cystitis increased BDNF mRNA and protein levels in L6 DRG. After CYP treatment for 48 h, the BDNF expression was examined in L6 DRG by immunohistochemistry (A-C) and real-time PCR (D). BDNF was expressed in small and medium sized sensory neurons (A, B). The average number of DRG neurons per unit area expressing BDNF was significantly increased post CYP treatment (C). The relative level of BDNF mRNA was also increased in L6 DRG during cystitis (D). Bar=80 µm. *, p<0.05 vs control. n=5-6 animals for each group.

At 48 h following intraperitoneal injection of CYP, BDNF expression was significantly increased in L6 DRG (Figure 1). BDNF immunoreactivity was expressed in small- to medium-sized DRG neurons with a number of 85.01 ± 10.75 cells per mm^2^ area in control animals (Figure 1A) and a number of 171.00 ± 21.27 cells per mm^2^ area in animals treated with CYP (Figure 1B), resulting in a 2-fold increase in cystitis when compared to control (Figure 1C, p<0.05). Real-time PCR examination showed that cystitis also caused an increase in BDNF mRNA level in the L6 DRG (Figure 1D).

**Figure 2:**
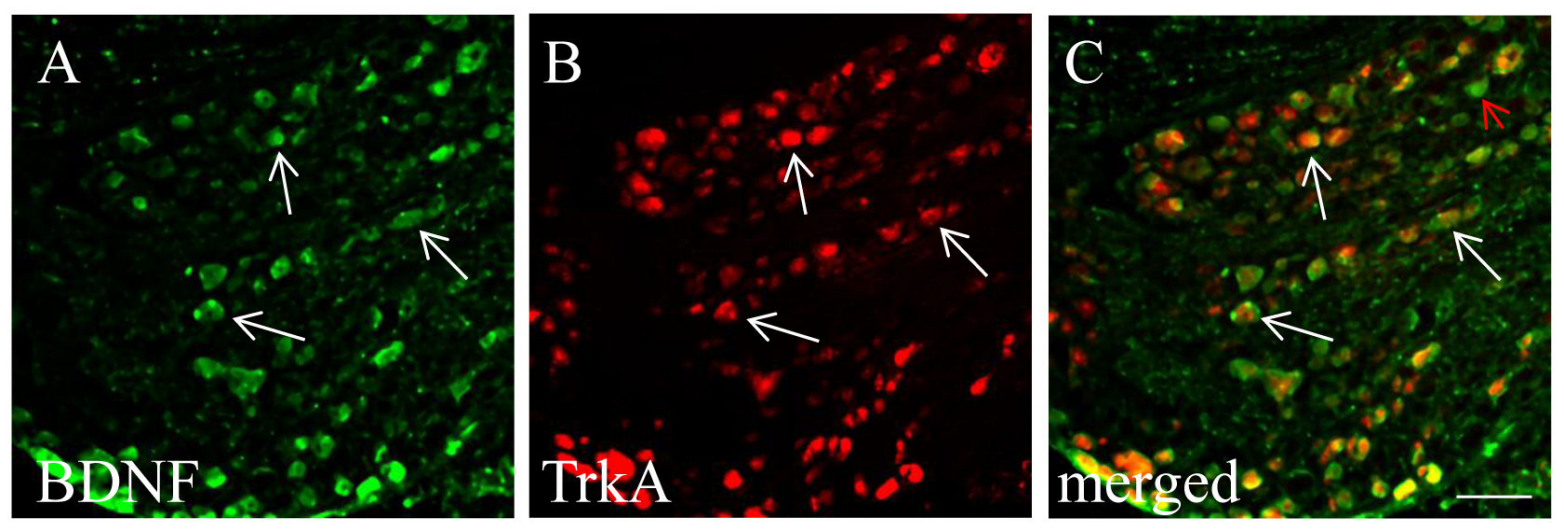
Co-localization of BDNF with TrkA in L6 DRG during cystitis. Double immunostaining showed that BDNF immunoreactivity (A, green staining) was co-localized with TrkA (B: red staining). Most of the BDNF positive neurons expressed TrkA (C: white arrows). A small number of BDNF positive cells did not express TrkA (C, red arrow). Bar = 60 µm. Three L6 DRGs from animals with cystitis were analyzed and consistent results were achieved.

**Figure 3:**
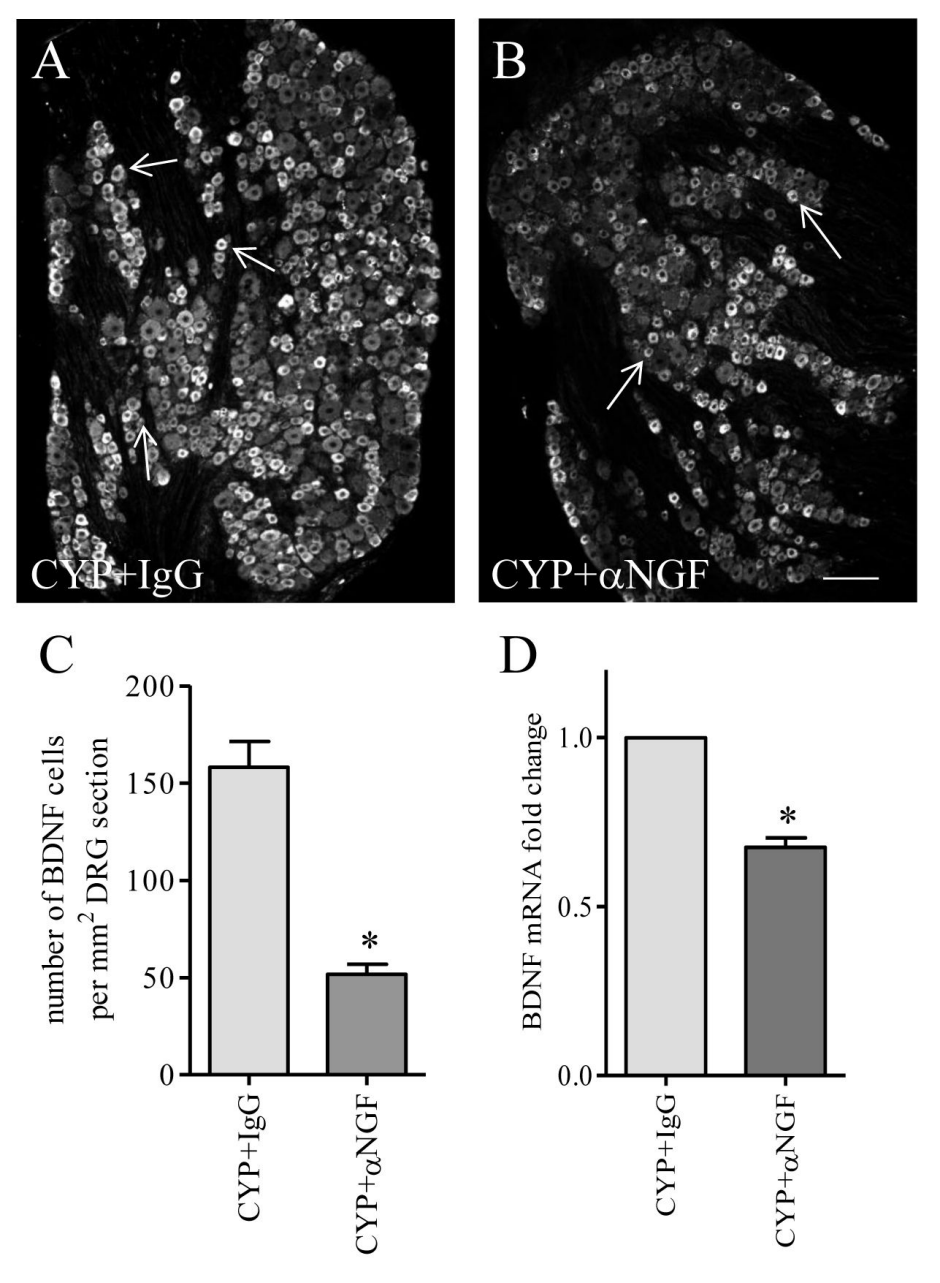
NGF immuno-neutralization attenuated cystitis-induced BDNF expression in L6 DRG. The number of BDNF immunoreactive neurons was significantly higher in L6 DRG from animals treated with CYP and control IgG (A, C) when compared to those from animals treated with NGF neutralizing antibody and CYP (B, C). Blockade of NGF activity in cystitis animals also reduced BDNF transcription in the L6 DRG (D). *, p < 0.05 vs CYP+IgG. n=5 animals for each group. Bar=80 µm.

It was reported that BDNF immunoreactivity was expressed in TrkA-positive neurons in DRG [50,51], and BDNF expression was able to be induced by exogenous NGF in vivo and in culture [43,51]. This was also true with cystitis. At 48 h post CYP treatment, NGF was increased in the urinary bladder [37], and BDNF immunoreactivity (Figure 2A, green cells) was largely expressed in TrkA-positive neurons (Figure 2B, red cells) in L6 DRG (Figure 2, white arrows indicate neurons co-expressing BDNF and TrkA). To examine whether cystitis-induced BDNF up-regulation in L6 DRG was triggered by endogenous NGF in vivo, we administered a NGF neutralizing antibody to rats with cystitis to block NGF action. Cystitic animals receiving the same amount of control IgG served as comparison. After 48 h post drug treatment, we found that the number of L6 DRG neurons expressing BDNF immunoreactivity was significantly decreased in animals receiving NGF neutralizing antibody when compared to animals receiving control IgG treatment (Figure 3A-C). Treatment with NGF neutralizing antibody also decreased the BDNF mRNA level in CYP-treated animals when compared to CYP+IgG treatment (Figure 3D), suggesting that endogenous NGF elicited BDNF transcription in the L6 DRG during cystitis. 

### Cystitis increased Akt activation in L6 DRG which was blocked by NGF neutralization

**Figure 4:**
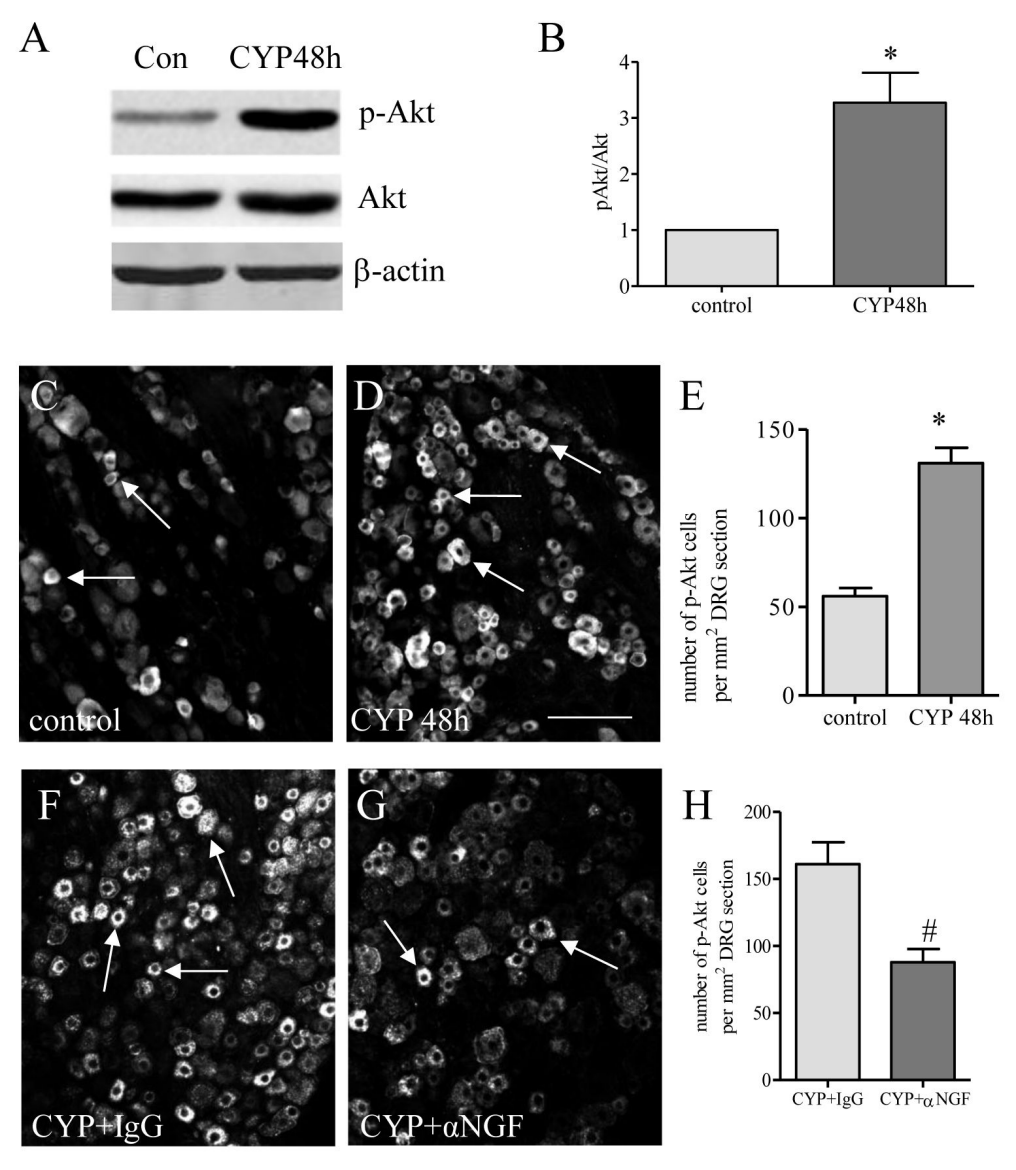
Cystitis-induced Akt activation in L6 DRG was blocked by NGF neutralization. Following CYP treatment, the Akt phosphorylation level (p-Akt) was significantly increased in the L6 DRG when examined by western blot (A, B). The result was confirmed with immunohistochemistry showing that the number of L6 DRG neurons expressing p-Akt was increased at 48 h post CYP treatment (C-E). NGF neutralization reduced cystitis-induced increases in the number of L6 DRG neurons expressing p-Akt (F-H). *, p < 0.05 vs control. n=5 animals for each group. Bar=80 µm.

Akt is one of the major downstream signaling components in NGF-initiated pathways. Western blot showed that the phosphorylation (activation) level of Akt (p-Akt) was increased in L6 DRG at 48 h post CYP injection (Figure 4A and B). Immunostaining showed that the number of neurons expressing p-Akt was also increased in L6 DRG examined at 48 h post CYP injection (Figure 4C-E). NGF neutralization in cystitis animals reduced Akt activity (Figure 4F-H) suggesting that activation of the Akt pathway was regulated by NGF in vivo. 

### PI3K-dependent Akt activation regulated BDNF expression in L6 DRG during cystitis

**Figure 5:**
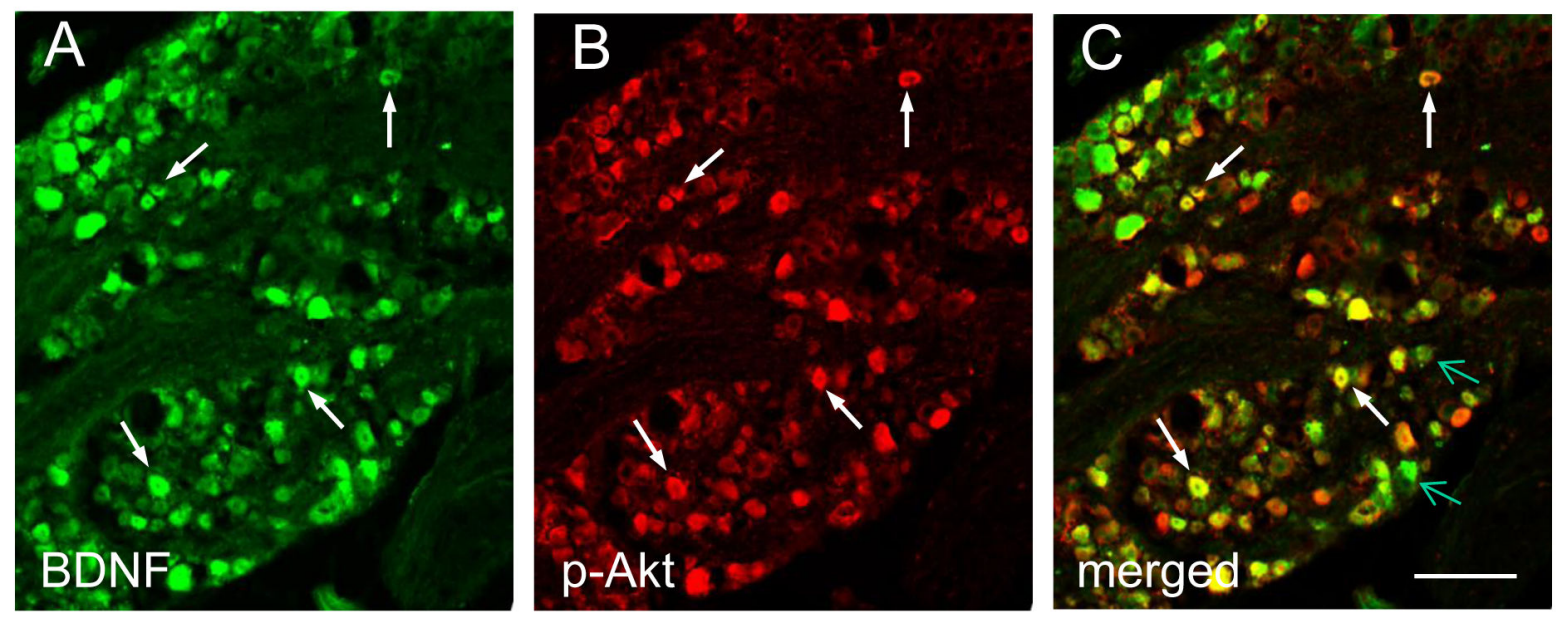
Co-localization of BDNF with p-Akt in L6 DRG during cystitis. Double immunostaining showed that a subpopulation of BDNF immunoreactive cells in L6 DRG during cystitis (A, green staining, white arrows) was co-localized with p-Akt (B: red staining). A number of BDNF positive cells (C, green arrows) did not express p-Akt. Bar = 60 µm. Five L6 DRGs from animals with cystitis were analyzed and consistent results were achieved.

We showed above that endogenous NGF increased BDNF expression in L6 DRG during cystitis (Figures 1 and 3), and NGF also activated Akt in L6 DRG (Figure 4). To examine whether NGF-induced Akt pathway had a role in NGF-induced BDNF expression during cystitis, we first compared the expression pattern of BDNF and p-Akt. We found that BDNF immunoreactivity (Figure 5A, green cells) was co-localized with p-Akt (Figure 5B, red cells) in L6 DRG in cystitis. Note that not all BDNF cells expressed p-Akt (Figure 5C, green arrows); this may be due to activation of other pathways such as the ERK pathway that also regulates BDNF expression upon NGF treatment [43]. 

**Figure 6:**
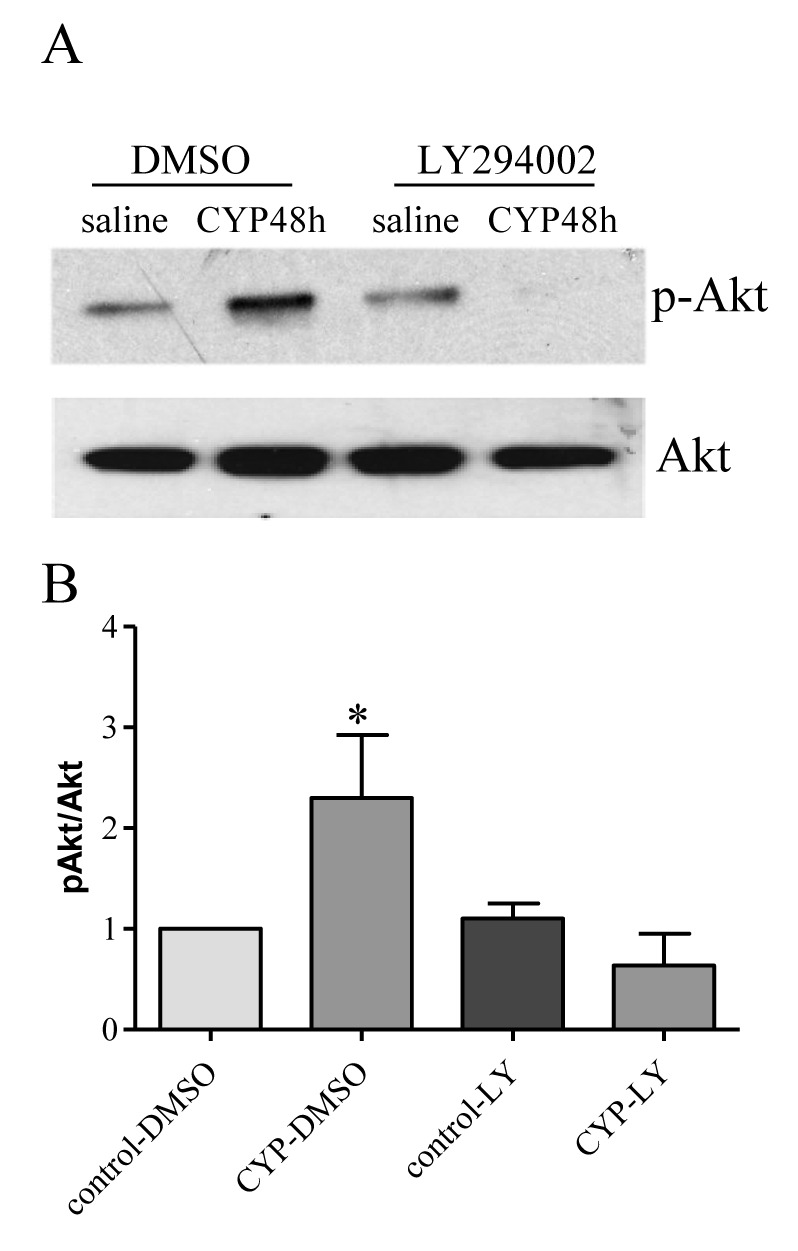
Cystitis-induced p-Akt was blocked by PI3K inhibitor LY294002. In vehicle (DMSO) treated animals, cystitis increased p-Akt levels in L6 DRG at 48 h post CYP treatment. In LY294002 treated animals, cystitis failed to induce Akt activation in the DRG. n= 3 for each experimental group. *, p < 0.05 vs control.

**Figure 7:**
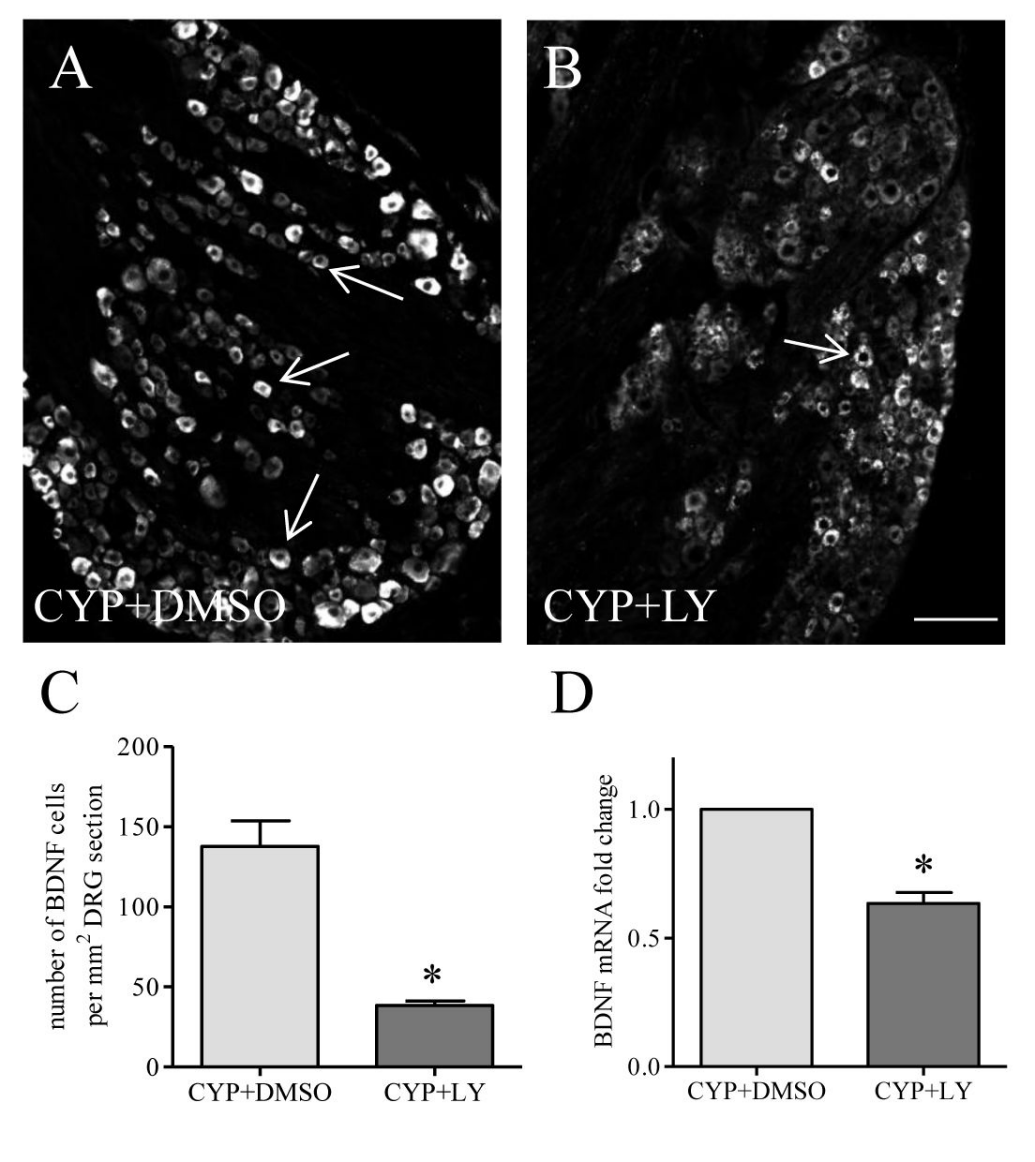
Effects of LY294002 on BDNF expression in L6 DRG during cystitis. At 48 h post drug treatment, the number of BDNF-positive cells in L6 DRG was significantly lower in CYP+LY294002 group than those from CYP+vehicle treated animals (A-C). LY294002 treatment also reduced BDNF transcriptional level when compared to vehicle control (D). *, p<0.05 vs control. Bar = 80 µm.

Akt can be activated in a PI3K-dependent or independent fashion [52,53,54]. In this study, we found that cystitis-induced Akt activation was blocked by the PI3K inhibitor LY294002 (Figure 6). In this treatment regimen, we found that prevention of endogenous Akt activity by LY294002 also blocked cystitis-induced BDNF protein (Figure 7A-C) and mRNA (Figure 7D) up-regulation in L6 DRG. 

### PI3K/Akt pathway mediated NGF-induced BDNF expression in DRG

**Figure 8:**
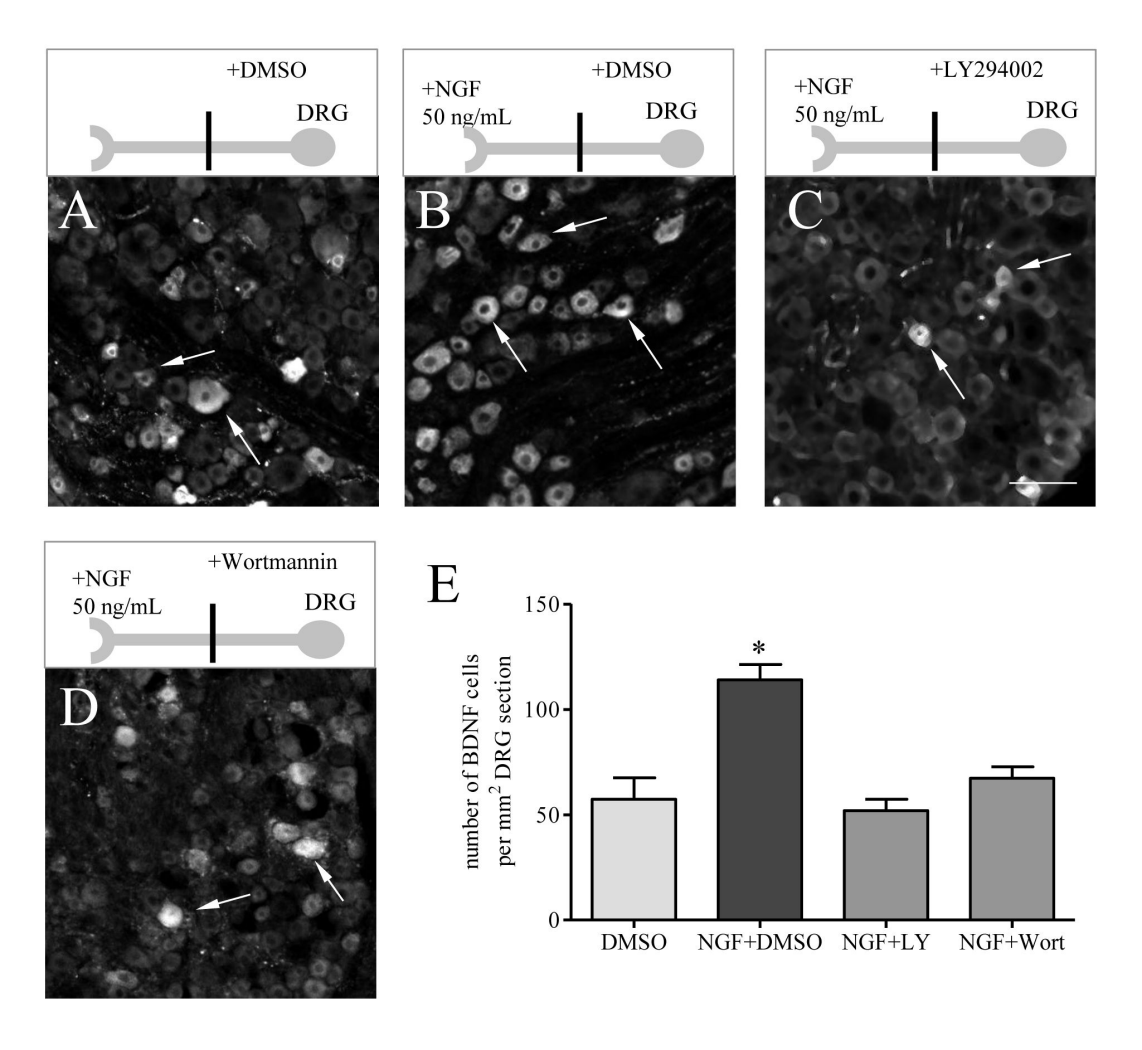
Retrograde NGF increased BDNF expression in sensory neurons, which was mediated by the PI3K/Akt pathway. In two-compartmented DRG-nerve culture, NGF (50 ng/mL) was added to the chamber containing the sensory axonal terminals. The ganglia were pre-treated with specific PI3K inhibitors LY294002 and Wortmannin, or vehicle. At 12 h after treatment, NGF increased the number of DRG neurons expressing BDNF (compare B to A) which was reversed by LY294002 treatment (compare C to B), and also by Wortmannin treatment (compare D to B). Histogram (E) showed summary results from 4 independent experiments. Bar= 40 µm. *, p<0.05 vs DMSO treatment.

 So far we have found that BDNF expression was regulated by endogenous NGF (Figures 1 and 3) and also by PI3K-dependent Akt activation in L6 DRG during cystitis (Figures 6 and 7). Endogenous NGF also regulated Akt activation in L6 DRG (Figure 4). To examine whether NGF-induced BDNF up-regulation was mediated by the PI3K/Akt pathway, we utilized an ex vivo culture system by applying exogenous NGF (50 ng/mL) to the nerve terminals of the DRG neurons in a two-compartmented L6 DRG-nerve preparation and examined the effect of retrograde NGF and the inhibition of Akt activity on BDNF expression in the DRG. This system was chosen based on previous findings by us and others that NGF was elevated in the inflamed urinary bladder [37,38] and its retrograde signal had a crucial role in mediating the target tissue-neuron interaction [55]. In this system, we found that NGF was able to enhance BDNF expression in the DRG neurons (Figure 8, compare B to A), which was blocked by the PI3K inhibitors LY294002 (compare Figure 8C to B) and Wortmannin (compare Figure 8D to B). 

## Discussion

BDNF is synthesized in the primary afferent neurons and plays a significant role in inflammation-induced afferent sensitization. To investigate the underlying mechanisms and pathways that regulate BDNF expression in the sensory neurons, we utilized a visceral inflammatory model with cystitis that was induced by intraperitoneal injection of CYP. We found that activation of the PI3K/Akt pathway led to BDNF up-regulation in the DRG in cystitis. The PI3K-Akt-BDNF axis was regulated by endogenous NGF in cystitis and also by retrograde NGF signaling in culture. In vivo, neutralization of NGF blocked the activity of Akt as well as the expression level of BDNF in DRG neurons, and the PI3K inhibition abrogated the Akt activity and also reduced BDNF level in the DRG. In an ex vivo ganglia-nerve two-compartmented preparation, application of NGF to the nerve terminals enhanced BDNF expression in the neuronal cell body, which was blocked by the PI3K inhibitors LY294002 and Wortmannin. These results suggested that up-regulation of BDNF in the primary afferent neurons during cystitis was regulated by NGF-induced PI3K/Akt activation in the DRG. 

BDNF is well recognized for its role in modulating central neuroplasticity and long-term memory [56]. Recent studies have revealed that BDNF also acts as a pain neuromodulator in inflammation- and injury-induced sensory hypersensitivity [31,57,58]. BDNF level is found to increase in primary afferent neurons in several models of inflammatory and neuropathic pain such as formalin and carrageenan-induced peripheral pain models or colitis-induced visceral pain [12,57,58]. In the present study, we show that BDNF mRNA and protein levels are also increased in cystitis, a painful inflammatory condition of the urinary bladder. In L6 DRG that receives sensory input from the urinary bladder, BDNF is mainly expressed in small- to medium-sized DRG neurons; this BDNF expression pattern is in agreement with those in other pain model systems [12,59]. Our recent study in a colitis-induced visceral pain model shows that BDNF is largely co-expressed with the transient receptor potential cation channel TRPV1 [15], suggesting a role of BDNF in nociception. In terms of bladder hyperactivity, we recently showed that intrathecal sequestration of BDNF action reduced colitis-induced bladder hyperactivity suggesting the role of BDNF in the regulation of bladder function [15]. Studies in other pain models also show a modulatory role of the BDNF system [16,57,60]. In a recent study in CYP-induced cystitis, Frias et al. show that blockade of BDNF action with TrkB-IgG reverses cystitis-induced bladder hyperactivity providing direct evidence that BDNF participates in bladder sensory hyperactivity during cystitis [31]. 

To better understand the regulatory mechanism of BDNF in the DRG, several experiments have been performed in vivo and in vitro. Apfel et al. [51] have characterized that 12 h after subcutaneous injection of recombinant human (rh) NGF to rat elicits BDNF mRNA level in the cervical DRG. Direct injection of NGF into the L4/5 nerve roots also increases BDNF expression in the DRG and produces mechanical allodynia [61]. In cultured DRG, exogenous NGF is able to induce BDNF transcript and sequestration of NGF blocks prostaglandin E2 (PGE2)-induced BDNF expression [62,63]. In the present study we show that sequestration of endogenous NGF in a cystitis rat model also blocks BDNF mRNA and protein levels in the DRG supporting a role of NGF in regulating BDNF expression in vivo. During cystitis or other visceral/peripheral inflammation, NGF is produced in the inflamed organ [25,37,55]. This will require retrograde transport of NGF signaling in order to regulate BDNF expression in the DRG. In our previous study [43] as well as in the current study, we show that application of NGF to the DRG nerve terminals facilitates BDNF expression in the DRG. Thus it is likely that in cystitis the elevated level of NGF in the inflamed urinary bladder can increase BDNF expression in the DRG through retrograde transport. The retrograde NGF action on affecting bladder sensory activity has been demonstrated by injection of exogenous NGF into the normal rat bladder which results in bladder hyperactivity [64]. Retrograde TrkA transport from visceral organs to the primary afferent neurons has also been seen in rats [55]. During cystitis, the expression level of TrkA is increased in bladder afferent neurons [49]. Increases in the TrkA level in these DRG neurons can enhance the responsiveness of these neurons to NGF thereby increasing NGF-initiated signal transduction, promoting BDNF expression in these neurons. In DRG, BDNF mRNA is expressed by numerous TrkA cells but by few TrkB or TrkC cells [50,65]. This suggests that BDNF is mainly synthesized in the sensory neurons that are responsive to NGF but not to other neurotrophins. 

Two major pathways are involved in NGF retrograde signaling and are activated in the neuronal cell bodies upon NGF retrograde stimulation. The ERK5 activation in the DRG by retrograde NGF has been demonstrated to have a role in BDNF expression [43]. The other major elements downstream of NGF retrograde signaling are the PI3K/Akt pathways. In this study, we show that the activity of Akt is increased in the L6 DRG during cystitis in a PI3K-dependent manner. Activation of Akt in the DRG also has an essential role in pain behavior induced by capsaicin [66]. Once Akt is activated, it can regulate BDNF expression in the DRG; in turn, anterograde transport of BDNF to the sensory terminals in spinal dorsal horn facilitates pain sensation [17,31,57]. In cystitis, BDNF action in the spinal cord induces ERK activation [31]; the latter has been demonstrated to increase in the spinal cord and has roles in cystitis-induced bladder hyperactivity [67,68]. BDNF may also modulate central sensitization through other mechanisms such as activation of the NMDA system [58] through activation of PKC [69]. The other pathway that is likely involved in BDNF up-regulation in the DRG during cystitis is the ERK5 pathway. ERK5 is essential in NGF retrograde transport and is activated in the neuronal somata [39]. After CYP treatment, ERK5 but not ERK1/2 is activated in the DRG suggesting a possibility that NGF retrograde signaling plays a role in primary afferent neuronal activation during cystitis [68]. Our recent study with DRG explants-nerve two-compartmented culture also shows that NGF-induced BDNF up-regulation in DRG neurons is reduced by a specific ERK5 inhibitor BIX02188 [43]. In DRG a subpopulation of BDNF expressing cells also express phospho-Akt, suggesting that BDNF expression is regulated by convergence of multiple pathways including Akt and ERK5. It is not clear whether ERK5 and Akt participate in BDNF up-regulation in a parallel or inter-dependent fashion. Our previous studies suggest that ERK5 and Akt are unlikely expressed in the same neurons in DRG [43]. 

 Cystitis is accompanied with increased urinary urgency, frequency and suprapubic and pelvic pain. Emerging evidence shows that inflammatory mediators generated in the urinary bladder triggers bladder sensory activation, thereby contributing to bladder hyperactivity [1]. Following CYP treatment, a number of inflammatory mediators are produced and released into the lamina propria where they sensitize the sensory nerve terminals and cause sensory hypersensitivity. The present study along with previous publications demonstrates that NGF is a critical endogenous mediator in regulating sensory plasticity by activating a series of signal transduction pathways and production of neuropeptides. BDNF produced by the sensory neurons that is regulated by the NGF signaling further facilitates spinal plasticity by reinforcing the activity of excitatory neuronal pathways in the primary sensory reflex. Considering the fact that NGF is also seen in IC patients, targeting of the neurotrophin system is essential in treatment of this painful disease. 
